# Developing a Referral Protocol for Community-Based Occupational Therapy Services in Taiwan: A Logistic Regression Analysis

**DOI:** 10.1371/journal.pone.0148414

**Published:** 2016-02-10

**Authors:** Hui-Fen Mao, Ling-Hui Chang, Athena Yi-Jung Tsai, Wen-Ni Huang, Jye Wang

**Affiliations:** 1 School of Occupational Therapy, College of Medicine, National Taiwan University, Taipei, Taiwan; 2 Department of Occupational Therapy, Institute of Allied Health Sciences, College of Medicine, National Cheng Kung University, Tainan, Taiwan; 3 Institute of Allied Health Sciences, College of Medicine, National Cheng Kung University, Tainan, Taiwan; 4 Department of Occupational Therapy, Kaohsiung Medical University, Kaohsiung, Taiwan; 5 Department of Physical Therapy, I-Shou University, Kaohsiung, Taiwan; 6 Department of Health Care Administration, Chang Jung Christian University, Tainan, Taiwan; University of Otago, NEW ZEALAND

## Abstract

Because resources for long-term care services are limited, timely and appropriate referral for rehabilitation services is critical for optimizing clients’ functions and successfully integrating them into the community. We investigated which client characteristics are most relevant in predicting Taiwan’s community-based occupational therapy (OT) service referral based on experts’ beliefs. Data were collected in face-to-face interviews using the Multidimensional Assessment Instrument (MDAI). Community-dwelling participants (*n* = 221) ≥ 18 years old who reported disabilities in the previous National Survey of Long-term Care Needs in Taiwan were enrolled. The standard for referral was the judgment and agreement of two experienced occupational therapists who reviewed the results of the MDAI. Logistic regressions and Generalized Additive Models were used for analysis. Two predictive models were proposed, one using basic activities of daily living (BADLs) and one using instrumental ADLs (IADLs). Dementia, psychiatric disorders, cognitive impairment, joint range-of-motion limitations, fear of falling, behavioral or emotional problems, expressive deficits (in the BADL-based model), and limitations in IADLs or BADLs were significantly correlated with the need for referral. Both models showed high area under the curve (AUC) values on receiver operating curve testing (AUC = 0.977 and 0.972, respectively). The probability of being referred for community OT services was calculated using the referral algorithm. The referral protocol facilitated communication between healthcare professionals to make appropriate decisions for OT referrals. The methods and findings should be useful for developing referral protocols for other long-term care services.

## Introduction

To quickly and accurately identify patients who will benefit from a specific treatment or intervention has always been challenging [1, 2]. Rapid increases in healthcare costs make it necessary to screen clients to efficaciously allocate limited healthcare resources. This is emphasized not only in the medical system but also in the long-term care (LTC) system to ensure the equitable screening and targeting of client needs.

As the number of older people and people with disabilities who need LTC services increases, community-based services continue to hold a predominant position among care services to achieve the goal of “aging in place” worldwide. Taiwan is one of the fastest aging countries in the world. The elderly (≥ 65 years old) population exceeded 1.49 million (7% of the total population) in 1993 and 2.9 million (12% of the total population) in 2015. It is estimated that there will be 4.73 million (20%) elderly in 2025 and that Taiwan will become a super-aged society [3]. According to national statistics[4], of the 0.76 million Taiwanese estimated to need LTC, most (63.2%) are elderly. Because of the fast-growing aging population, the needs and demands for LTC services have become urgent and significant.

Community LTC services include a broad set of health, personal care, and supportive services that meet the needs of people of all ages whose capacity for the activities of daily living (ADLs) are limited because of physical or mental disorders [5]. LTC can be provided in a variety of settings. The recently announced Patient Protection and Affordable Care Act also defines and provides coverage for the USA's LTC services; it declares that “Most long-term care is provided at home. Other kinds of LTC services and support are provided by community service organizations and in LTC facilities" [6]. Community rehabilitation services, such as occupational therapy (OT), physical therapy (PT), and speech therapy, are provided at clients’ homes, community centers, group homes, and assisted-living communities, and are integral parts of LTC. The goal of OT services in LTC is to help people with physical disabilities, mental disabilities, or both, increase their independence in ADLs and community living skills, and to prevent functional deterioration by enabling them to participate in meaningful, everyday activities [7, 8]. The following are the primary components of community-based OT services: 1) Restorative care & ADL assessments to promote a client’s ability to function to their full potential, thereby increasing their independence and self-esteem: specific training includes BADLs, IADLs, and leisure activities, visual perception, hand function, communication, interpersonal skill, range of motion, muscle strength, balance training, cognitive retraining, habit training, life-style reconstruction, and evaluating and fabricating splints; 2) Compensatory approaches: strategies include adaptive equipment services with need assessment, prescription and education on proper use, and environmental modifications; 3) Educational approaches: teaching clients and caregivers about skills important for client safety, participation, dignity, and independence [4, 9]. The increase in independence usually reduces the burden of personal care and costs of health and welfare services and improves the client’s quality of life (QoL) [10, 11]. In the present study, we define the community-based OT service in accordance with governmental regulations in Taiwan and include OT services provided at the clients’ homes or day service centers (such as adult day care centers, community rehabilitation centers for people with physical or mental dysfunctions, etc.), but not in nursing homes or residential care communities.

In the last several decades, LTC services in Taiwan have been evolving to meet the society’s changing demographics and demands to provide care for people with disabilities. As a result, the majority of people with disabilities receive LTC services; however, they often have to go through lengthy and complicated application procedures to get LTC services. In the past, LTC services in Taiwan were managed by three governmental systems (the healthcare, social welfare, and veteran administrations), all of which had different application processes and benefit packages. Therefore, the LTC services were not properly integrated and the care was often fragmentary [12, 13]. For example, home modification and assistive devices can be covered by different agencies and funding sources, depending upon the client’s diagnosis, age, economic status, type of housing and reason for disability, and upon the types of equipment required. To solve these problems and to be responsive to the increasing demands for LTC services, the Taiwan government proposed the 10-Year Long-term Care Plan in 2007 [13]. The overall goal is to establish an LTC system, especially community-based programs, to provide integrated and appropriate services for people ≥ 65 years old with mental disabilities, physical disabilities, or both. Since 2008, every city and county has implemented the 10-year Long-term Care Plan and has begun to integrate local community LTC services. The scope of the 10-year Long-term Care Plan services includes eight domains: (1) home helper services, (2) home nursing, (3) home and community-based rehabilitation (OT and PT), (4) respite care, (5) meal services, (6) reimbursement and rental of assistive devices and medical auxiliaries not covered elsewhere, environmental modifications, (7) transportation services, and (8) institutional LTC services [13, 14].

The 10-year Long-term Care Plan required all cities and counties to set up their own LTC management centers for resource integration and allocation. Eligibility was determined first based on the age and the extent of ADL or IADL dependence within the past six months [13, 14]. If the client was eligible, the care managers would conduct an in-person assessment using a comprehensive long-term care needs assessment tool, similar to the assessments commonly used in North America and Europe, such as the interRAI Home Care Assessment System, which is mandated for use with home care clients in some areas to inform and guide comprehensive care and service planning [15–17]. After finishing the assessment, the care managers initially determine whether and which of the LTC services are needed, develop a care plan, and make referrals for the services (such as home OT service). However, the care managers often have to determine the need for OT services based on a very minimal criteria (e.g., home-bound, ADL or IADL dependence, etc.), which may lead to an inconsistent referral pattern for OT and result in poor control of already limited resources.

Taiwan’s legislature passed a Long-Term Care Services Act in 2015. A Long-Term Care Insurance Act is expected to be enacted in the near future [18]. LTC insurance will likely cover people in all age groups with physical or mental disabilities (chronic mental disorders, dementia, and intelligence dysfunctions), or both. Therefore, the scope of OT services covered by LTC insurance will expand. In 2011, the Ministry of Health and Welfare started a series of research projects to develop the various services included in LTC. A committee of leading experts and government officials in charge of drafting government LTC insurance in Taiwan [19] envisioned an assessment tool that assists care managers to evaluate the LTC service needs of people with various disabilities and to allocate needed services. Hence, the Multidimensional Assessment Instrument (MDAI) was developed after a series of research projects that evaluated its feasibility. The Taiwan Occupational Therapy Association was charged with developing an empirically tested screening model that would facilitate effective community-based LTC OT referrals. The part of the processes and results of this project are reported here.

The problem with rehabilitation referrals has been widely documented in the literature. In the medical system and in the LTC system, they are usually determined by physicians, care managers, or other allied health professionals in a multidisciplinary team [14, 20]. The levels of rehabilitation referrals have frequently been considered inadequate because many people who need rehabilitation are not referred to the service. A low level of knowledge about therapy, uncertainty about its effectiveness, access limitations, and a lack of perceived needs for therapy have been identified as barriers to rehabilitation referrals[15, 21–23]. It appears that there was a discrepancy between the professional view and actual practice about which clients with what characteristics required rehabilitation services. According to a Canadian study [23], close to three-quarters of home-care clients who have been identified as having rehabilitation potential do not receive any type of rehabilitation therapy. A 2011 government report on the 10-year Long-term Care Plan concluded that close to one-half of LTC clients who have been identified by care managers as having rehabilitation needs do not receive any type of services [24]. This might be attributable to a lack of understanding by some clients of how rehabilitation services can help them, and it might also be the result of a lack of well-defined referral criteria. This lack of agreement between the referrer and the professionals who provide the services is a problem because it means that the referral status and service needs might be different when different people decide on referrals. Most important, however, is that professionals are unable to provide their services to the clients who they believe will benefit most. Active promotion of therapy and knowledge of its effectiveness were identified as facilitators for successful referrals [25]. Therefore, a protocol that predicts referral for rehabilitation in an LTC system with limited resources is essential.

To the best of our knowledge, there are few referral protocols for care managers to determine who might benefit from rehabilitation services. The interRAI Contact Assessment [26] was created using a subset of interRAI Home Care Reporting System items as a preliminary assessment of home-care clients to identify those who require comprehensive assessment and to identify their needs for a number of services, including rehabilitation. A recent study [27] in Canada developed a rehabilitation algorithm and confirmed the importance of functional decline and mobility variables for targeting rehabilitation services. This is the first study that sought to understand whether an algorithm can be used to assist the referral process of home-based rehabilitation. However, the study’s participants were restricted to those ≥ 65 years old and for home-based PT, OT, or both. Our study included broader criteria, such as people who were < 65 years old. As the needs for rehabilitation in LTC settings increase, there is still no screening tool for care managers to determine who would benefit from community-based LTC OT services. Additional studies in a variety of contexts are required to bridge the gap between a need for services and their provision-and-use, especially while developing the LTC system.

Although it is still uncertain which kind of OT services will be reimbursed by LTC insurance or other payment resources (e.g., community-based OT services for psychiatric diseases are partially covered by Taiwan’s National Health Insurance program. The uniqueness of the present study is that it identifies clients who need and will likely benefit from OT services based on the judgments of OT professionals. This is different from other studies that identify predictors of rehabilitation use based on utilization data, which might be biased because of local regulations. The goal of our study was to develop a referral protocol based on the MDAI to predict which clients need and will likely benefit from home- and community-based OT services according to the judgments of OT professionals.

## Methods

### Study participants

The institutional review board at Kaohsiung Medical University approved this study. Seven hundred sixty-four potential community-dwelling participants were randomly selected (stratified by age, gender, and geographical location) from the 10,015 participants of the 2010 wave National Survey of Long term Care Needs in Taiwan [28]. The inclusion criteria of the 2010 study were: (1) ≥ 18 years old, (2) a Barthel Index (BI) of an ADL score ≤ 70, or a BI > 70 [29] combined with one of the following criteria: Lawton Scale ≤ 2 [30], a Short Portable Mental Status Questionnaire (SPMSQ) ≥ 6 [31], or a government-issued disability certificate. Those who were not targets for OT services because they were unlikely to benefit from them were excluded from the study. The exclusion criteria mandated by the government were having hearing impairments, major organ dysfunctions, or both, but having intact BADL and IADL functions.

### Assessment tools

The MDAI was developed by a committee of leading experts and government officials who were in charge of drafting government LTC insurance in Taiwan [19]. The goal of the MDAI was to assist care managers evaluate the LTC service needs of people with various disabilities and to allocate needed services. The MDAI includes six domains: (1) ADLs and IADLs, (2) communication abilities (consciousness, hearing and visual functions, the ability to understand, and the ability to express oneself), (3) special care needs (disease history, skin conditions, medications, pain, muscle strength, joint ROM, balance and ambulation, history of falling, using assistive devices and specialized care, i.e., nasogastric tubes, catheters, etc.), (4) cognitive, emotional, and behavioral problems, (5) home environment, family, and social support, and (6) caregiver burden. This study considered only variables relevant to community-based OT referral.

ADLs were measured using the ten-item BI (score range: 0–100; a higher score means better ADL performance) [29]. IADLs were measured using the eight-item Lawton Scale (score range: 0–8; a higher score means better IADL performance) [30]. The disease history included physicians’ diagnoses of psychiatric, neurological, and musculoskeletal disorders. Cognitive function was measured using the ten-item SPMSQ (score range: 0–10; a higher score means better cognitive function) [31]. Behavioral and emotional problems were measured using fourteen questions chosen from the Behavioral Pathology in Alzheimer's Disease Scale (BEHAVE-AD) [32] and the Cohen-Mansfield agitation inventory (CMAI) [33]. Any item present during the previous 3 months is scored “1”. The score range is 0–14; a higher score means more severe behavioral and emotional problems. Caregiver burden was measured using the 13-item Caregiver Strain Index [34] (score range: 0–13; a higher score means a higher caregiver burden). Some of the detailed descriptions are listed at the end of the tables.

The MDAI was completed in 40–60 minutes by interviewers trained to collect information based on standardized procedures. The majority of the items were based on self-reported information from clients or proxies and caregivers. Some items were obtained through observation (e.g., sitting and standing balance, pressure sores, etc.). The MDAI has a very high content validity index (CVI > = 0.90) in all 6 domains and excellent inter-rater and test-retest reliabilities (r > 0.90) [19].

### Data collection

The 764 potential participants were invited by telephone to join the study. Fifty-eight had died since 2011, 253 refused to participate, 76 had been admitted to institutions, 152 were lost to follow-up, and 4 were excluded because of severe hearing impairment or an inability to communicate. The remaining 221 participants or their proxies signed a written informed consent form and completed the structured face-to-face MDAI interviews. The interviewers experienced in comprehensive LTC care needs assessments were given one additional day of MDAI assessment training before the fieldwork and were regularly audited to ensure that the assessment was correctly completed. All interviews were conducted from June through October 2013.

Two occupational therapists experienced in LTC independently reviewed the completed MDAI documents and were instructed that their primary goal was to determine whether each study participant needed and was likely to benefit from OT services. Before the interviewers began to collect data, we held a series of 4 focus groups of occupational therapists and experts experienced with providing services in LTC settings. The group reached a consensus on 3 categories of criteria for OT referral of therapy services. The first category was clients with functional deficits or barriers to achieving independence or community integration (e.g., recent function decline; needing assistance with ADLs; home environment barriers that affect mobility or safety; poor social participation or life arrangement; behavior and emotional problems; etc. The second category was clients with potential to make significant gains because they have learning potential and are able to follow instructions. The third category was clients who need intervention to prevent additional rapid deterioration or secondary problems (e.g., frequent falls, unskilled caregivers, etc.). The two therapists attended all the group meetings and were familiar with the consensus. They then used their best clinical judgment to evaluate the results of MDAI assessments and make referral decisions. If the recommendation of these two therapists differed, a third senior therapist joined the discussion to reach a consensus. Thirty-four participants were also randomly selected and visited by an experienced therapist and an interviewer. The therapist’s recommendations from these in-person visits were used to validate those from the MDAI reviews. The results between in-person therapist visits and MDAI record reviews by the therapists were fairly consistent. The two raters agreed on 30 of 34 (88.2%) of the referrals. The κ value was 0.765.

### Statistical analysis

R-3.0.2 (http://cran.r-project.org/bin/windowsbase/old/3.0.2/) [35] was used for all statistical analysis. Significance was set at *p* ≤ 0.05 (two-sided). The two occupational therapists assigned all participants to one of two outcome groups: 0 = No need for OT, 1 = Need for OT. Of the 268 available MDAI items, only those relevant to OT were retained in the analysis. In univariate analysis, the Kruskal-Wallis test was used for differences in the distributions of continuous variables, and χ^2^ and Fisher’s Exact tests were used for the differences in the distributions of categorical variables between the two groups. A multivariate analysis was then completed by fitting logistic regression models to estimate the effects of predictors on whether a participant needed OT services. All of the relevant significant and non-significant univariate covariates (Tables 1 and 2), and some of their interactions, were put on the stepwise variable selection list. Specifically, 72 variables were put on the variable list for the IADL Model and 73 for the BADL Model.

**Table 1:** Demographic Characteristics of Participants.

	Total (n = 221) n (%)	No Need for OT referral (n = 50) n (%)	Need for OT referral (n = 171) n (%)	P-value
**Gender**				0.155
Male	122 (55.2)	32 (64)	90 (53)	
Female	99 (44.8)	18 (36)	81 (47)	
**Age (Years)**				0.008
**Mean (SD)**	63.64 (19.6)	56.74 (17.5)	65.65 (19.8)	0.003
20–35	29 (13.1)	9 (18.0)	20 (11.7)	
36–50	29 (13.1)	9 (18.0)	20 (11.7)	
51–65	51 (23.1)	17 (34.0)	34 (19.9)	
66–80	63 (28.5)	12 (24.0)	51 (29.8)	
> 80	49 (22.2)	3 (6.00)	46 (26.9)	
**Marital Status**				0.005
Married / Cohabitating	102 (46.2)	33 (66.0)	69 (40.4)	
Divorced / Separated	12 (5.4)	4 (8.0)	8 (4.7)	
Widowed	53 (24.0)	3 (6.0)	50 (29.2)	
Single	54 (24.4)	10 (20.0)	44 (25.7)	
**Education**				0.001
Illiterate / Elementary school	117 (52.9)	18 (36.0)	99 (57.9)	
7th to 12th Grade	73 (33.0)	21 (42.0)	52 (30.4)	
Bachelor or Graduate Degree	19 (8.6)	10 (20.0)	9 (5.3)	
Special Education	12 (5.4)	1 (2.0)	11 (6.4)	
**Household Income**				0.116
Poverty level (≤ NT$ 10,869/mo)	5 (2.3)	3 (6.0)	2 (1.2)	
Near poverty level (NT$ 10,869–16,304/mo)	7 (3.2)	1 (2.0)	6 (3.5)	
Ordinary level (≥ NT$16,304/mo)	209 (94.6)	46 (92.0)	163 (95.3)	
**Living Status**				0.496
Living alone	7 (3.2)	2 (4.0)	5 (2.9)	
Living with family (others)	214 (96.8)	48 (96.0)	166 (97.1)	
**Home Barriers**				0.020
No	178 (80.5)	47 (90.4)	131 (77.5)	
Yes	43 (19.5)	5 (9.6)	38 (22.5)	

NT$: New Taiwan dollars (US$1 = ca. NT$31).

**Table 2:** Clinical Characteristics of Participants.

	Total (n = 221) n (%)	No Need for OT Referral (n = 50) n (%)	Need for OT Referral (n = 171) n (%)	P-value
**Consciousness Level**				< 0.001
Clear	167 (75.6)	50 (100.0)	117(68.4)	
Unclear	54 (24.4)	0 (0.0)	54 (31.6)	
**Ability to Express Oneself**				< 0.001
Good	124 (56.1)	44 (88.0)	80 (46.8)	
Fair	44 (19.9)	3 (6.0)	41 (24.0)	
Poor	53 (24.0)	3 (6.0)	29.2)	
**Ability to understand**				< 0.001
Good	123 (55.7)	45 (90.0)	78 (45.6)	
Fair	55 (24.9)	4 (8.0)	51 (29.8)	
Poor	43 (19.5)	1 (2.0)	42 (24.6)	
**Visual Function**				0.004
Normal	110 (49.8)	35 (70.0)	75 (43.9)	
Fair	62 (28.1)	7 (14.0)	55 (32.2)	
Poor	49 (22.2)	8 (16.0)	24.0)	
**Hearing**				0.005
Normal	135 (61.1)	39 (78.0)	96 (56.1)	
Impaired	86 (38.9)	11 (22.0)	75 (43.9)	
**SPMSQ Score (n = 148)**^a^^,^ ^e^				< 0.001
Mean (SD)	6.76 (2.8)	8.63 (1.69)	6.05 (2.81)	
**SPMSQ score 2–9** ^a^				0.0063
Yes	104(47.06)	15(30.0)	89(52.05)	
No	117(52.94)	35(70.0)	82(47.96)	
**Cognitive level**				< 0.001
Good (SPMSQ 8–10)	80(36.20)	36(72.00)	44(25.73)	
Mildly impaired (SPMSQ 6–7)	21(9.50)	2(4.00)	19(11.11)	
Moderately impaired (SPMSQ 3–5)	30(13.58)	2(4.00)	28(16.37)	
Severely impaired (SPMSQ 0–2, and NA)	90(40.72)	10(20.0)	80(46.78)	
**Perceptual Function (n = 163)** ^e^				0.016
Normal	142 (87.1)	42 (97.7)	100 (83.3)	
Impaired	21 (12.9)	1 (2.3)	20 (16.7)	
**Musculoskeletal Disorders (n = 220)** ^e^				0.068
No	168 (76.4)	43 (86.0)	125 (73.5)	
Yes	52 (23.6)	7 (14.0)	45 (26.5)	
**Neurological Disorders (n = 220)** ^b^^,^ ^e^				< 0.001
No	131 (59.5)	42 (84.0)	89 (52.4)	
Yes	89 (40.5)	8 (16.0)	81 (47.6)	
**Psychiatric Disorders (n = 220)** ^c^^,^ ^e^				0.003
No	142 (64.5)	41 (82.0)	102 (59.4)	
Yes	78 (35.5)	9 (18.0)	69 (40.6)	
**Dementia Diagnosis**				< 0.001
No	181(81.9)	49(98.0)	132(77.19)	
Yes	40(18.1)	1(2.0)	39(22.8)	
**Barthel Index**				< 0.001
Mean (SD)	73.19(34.881)	99.40 (2.399)	65.53 (36.219)	
**Lawton IADL Scale**				< 0.001
Mean (SD)	4.01 (3.164)	7.44 (1.053)	2.99 (2.850)	
**IADL decline recently**				0.3063
No	209(94.57)	49(98.00)	160(93.57)	
Yes	12(5.43)	1(2.00)	11(6.43)	
**BADL decline recently**				0.020
No	204 (92.3)	50 (100.0)	154 (90.06)	
Yes	17 (7.69)	0 (0.0)	17 (9.94)	
**Ambulation Function (n = 217)** ^e^				< 0.001
Normal	154 (71.0)	50 (100.0)	104(63.2)	
Some limitation	22 (10.1)	0 (0.0)	22 (12.9)	
Severe limitation	41 (18.9)	0 (0.0)	41 (24.0)	
**Joint Range of Motion Limitation**				< 0.001
No	138 (62.4)	47 (94.0)	91 (53.2)	
Yes	83 (37.6)	3 (6.0)	80 (46.8)	
**Sitting Balance**				< 0.001
Normal	182 (82.4)	50 (100.0)	132 (77.2)	
Poor	39 (17.6)	0 (0.0)	39 (22.8)	
**Standing Balance**				< 0.001
Normal	152 (58.8)	50 (100.0)	102 (59.6)	
Poor	69 (31.2)	0 (0.0)	69 (40.4)	
**Falling in the Past 6 Months**				< 0.001
No	169 (76.5)	49 (94.2)	115 (68.0)	
Yes	52 (23.5)	3 (5.8)	54 (32.0)	
**Fear of Falling (n = 190)**^e^				< 0.001
No	113 (59.5)	44 (88.0)	69 (49.3)	
Yes	77 (40.5)	6 (12.0)	71 (50.7)	
**Awareness of Risks in Daily life (n = 220)** ^e^				< 0.001
Yes	124 (56.4)	45 (90.0)	79 (46.5)	
No	96 (43.6)	5 (10.0)	92 (53.5)	
**Use of Assistive Devices**				< 0.001
No device	101 (45.7)	40 (80.0)	61 (35.7)	
Use with no difficulty	91 (41.2)	10 (20.0)	81 (47.4)	
Use with difficulty	29 (13.1)	0 (0.0)	29 (17.0)	
**Social Contact**				< 0.001
2–3 Times per week	100 (45.2)	11 (22.0)	89 (52.0)	
At least once per week	67 (30.3)	17 (34.0)	50 (29.2)	
Seldom or never	54 (24.4)	22 (44.0)	32 (18.7)	
**Hours Safe to Stay at Home Alone Perceived by Caregivers (n = 211)** ^e^				< 0.001
0	30 (14.2)	0 (0.0)	30 (18.2)	
<1	36 (17.1)	1 (2.2)	35 (21.2)	
1–3	35 (16.6)	4 (8.7)	31 (18.8)	
3–6	14 (6.6)	1 (2.2)	13 (7.9)	
6–9	17 (8.1)	3 (6.5)	14 (8.5)	
>9	79 (37.4)	37 (80.4)	42 (25.5)	
**Number of Behavioral and Emotional Problems (n = 211)** ^d^^,^ ^e^				
Mean (SD)	5 (8.339)	0.49 (2.191)	6.25 (8.973)	< 0.001
**≥ 2 Behavioral and Emotional Problems**				< 0.001
Yes	95 (42.99)	4(8.0)	91 (53.22)	
No	126 (57.01)	46(92.0)	80 (46.78)	
**≥ 3 Behavioral and Emotional Problems**				< 0.001
Yes	90 (40.72)	4(8.0)	86 (50.29)	
No	131 (59.28)	46(92.0)	85 (49.71)	
**Caregiver Strain Index**				
Mean (SD)	5.12 (4.309)	1.05 (1.66)	6.21 (4.145)	< 0.001
**Caregivers with disabilities**				0.957
No	194 (87.78)	44 (88.00)	150 (87.72)	
Yes	27 (12.22)	6(12.00)	21 (12.28)	
**Caregivers’ health**				0.053
Good	19(8.60)	6(12.0)	13(7.60)	
Fair	128(57.92)	32(64.0)	96(56.14)	
Poor	65(29.41)	8(16.0)	57(33.33)	
Missing	9(4.07)	4(8.0)	5(2.92)	

OT: occupational therapy; IADL: instrumental activities of daily living; SD: standard deviation.

^a^ SPMSQ: Short Portable Mental Status Questionnaire, range: 0–10; The cutoff points of 2 and 9 were determined using GAM analysis. There were only 148 respondents who were able to respond to SPMSQ; 73 participants who were unable to respond were coded as “0”.

^b^ Neurological disorders include: self-reported diagnoses of stroke, paraplegia, tetraplegia, Parkinson's disease etc.

^c^ Psychiatric disorders include: self-reported diagnoses of major depressive disorder, bipolar disorder, and psychosis, etc.

^d^ The cutoff point of 2 (for ADL model) and 3 (for IADL model) for Number of behavioral and emotional problems was determined using GAM analysis.

^e^ There were some missing values for certain variables, and the number of participants who responded to these questions is shown in parentheses.

The goal of the regression analysis was to find one or more parsimonious regression models that fit the observed data well enough to estimate the effect or predict the outcome. To ensure the quality of the regression analysis, basic model-fitting techniques for (1) variable selection, (2) goodness-of-fit (GOF) assessment, and (3) regression diagnostics and remedies were used. Stepwise variable selection (with iterations between the forward and backward steps) was used to obtain the best final logistic regression model candidate. The significance levels for entry (slentry) into the model and for staying (slstay) in the model were conservatively set at 0 and 0.15, respectively. Then, based on our substantive knowledge and clinical judgment, we individually and manually dropped the nonsignificant covariates (*p* > 0.05) until all regression coefficients were significantly different from 0, and thus identified the best final logistic regression model. Any discrepancy between the results of univariate analysis and multivariate analysis (Table 3) was likely due to the confounding effects of the uncontrolled covariates in univariate analysis. To evaluate the GOF of our fitted logistic regression model, we examined the estimated area under the receiver operating characteristic (ROC) curve (c-statistic), the adjusted generalized *R*^2^, and the Hosmer-Lemeshow GOF test.

**Table 3:** Multivariate Analysis of the Predictors of OT Referral by Fitting Multiple Logistic Regression Model: IADL and BADL Models.

	IADL Model			BADL Model		
	OR	P-value	95% CI	OR	P-value	95% CI
**Lawton Scale**	0.41	< 0.001	0.28–0.60	-	-	-
**Barthel Index**	-	-	-	0.77	< 0.01	0.64–0.94
**Dementia**	61.49	< 0.05	2.31–1636.29	48.26	< 0.05	2.09–1114.66
**SPMSQ (2–9)**	14.60	< 0.01	2.93–72.79	8.87	< 0.01	2.10–37.50
**ROM Limitation**	13.76	< 0.01	2.01–94.24	13.97	< 0.01	2.08–93.87
**Psychiatric Disorders**	11.04	< 0.01	2.17–56.09	13.95	< 0.001	3.12–62.34
**≥ 2 (for ADL model) or ≥ 3 (for IADL model) Behavioral and Emotional Measures**	9.87	< 0.01	1.79–54.35	5.78	< 0.05	1.25–26.81
**Fear of Falling**	11.45	< 0.01	2.15–61.11	13. 93	< 0.01	2.71–71.75
**Unable to Express Oneself**	-	-	-	6.85	< 0.05	1.23–38.04

OT: occupational therapy; IADL: instrumental activities of daily living; BADL: basic activities of daily living; OR: odds ratio; CI: confidence interval; SPMSQ: Short Portable Mental Status Questionnaire, range: 0–10; ROM: range of motion. **IADL Model:** Sample size (*n*) = 221 and the number of estimated parameters (*p*) = 8. Goodness-of-fit assessment: *n* = 221, adjusted generalized *R*^2^ = 0.800 > 0.3, the estimated area under the Receiver Operating Characteristic (ROC) curve = 0.977 > 0.7 (se = 0.008), the modified Hosmer-Lemeshow goodness-of-fit *F* test *p* = 0.5001 > 0.05 (df = 9, 211), and the ratio of residual deviance and its degrees of freedom, 71.5356/213 = 0.3358 ≤ 1.0, which indicated an excellent fit. **BADL Model:** Sample size (*n*) = 221 and the number of estimated parameters (*p*) = 9. Goodness-of-fit assessment: *n* = 221, adjusted generalized *R*^2^ = 0.778 > 0.3, the estimated area under the Receiver Operating Characteristic (ROC) curve = 0.972 > 0.7 (se = 0.009), the modified Hosmer-Lemeshow goodness-of-fit *F* test *p* = 0.6234 > 0.05 (df = 9, 211), and the ratio of residual deviance and its degrees of freedom, 78.1694/212 = 0.3687 ≤ 1.0, which indicated an excellent fit. In both logistic regression models, *n*–*p* ≥ 200, which indicated that our sample size was not too small.

Generalized additive models (GAMs) were fitted to detect the nonlinear effects of continuous covariates and to identify appropriate cutoff points for discretizing a continuous covariate, if necessary, during the stepwise variable selection procedure. Computationally, the VGAM function (with the default values of smoothing parameters) of the VGAM package for R (https://www.stat.auckland.ac.nz/~yee/VGAM/) [35, 36] was used to fit GAMs for each set of the binary responses of Yi. Finally, the statistical tools of regression diagnostics for residual analysis, detecting influential cases, and checking multicollinearity were used to discover any model or data problems. Values of the variance-inflating factor (VIF) ≥ 10 in continuous covariates or ≥ 2.5 in categorical covariates indicate the occurrence of the multicollinearity problem in some of the fitted logistic regression model covariates.

## Results

### Characteristics of Study Participants

Of the 221 participants, 171 (77.4%) were recommended for OT intervention (Table 1). They were more likely to be older, to be widowed or single, and to have less than a bachelor’s degree (Table 1). They also had more musculoskeletal, neurological, and psychiatric disorders; were more likely to have impaired vision, hearing, and cognitive function; and were more likely to be limited in BADLs and IADLs (Table 2). Those who were recommended for OT were more likely to have limited ambulation, limited joint ROM, poor balance function, environmental barriers at home, and a history of falling within six months. They had more severe behavioral and emotional problems, and their caregivers had significantly higher caregiver strain (Table 2).

### Predictors of OT referrals using BADL and IADL models

Bivariate analysis showed that BADL and IADL scores were highly correlated (r = 0.833). Thus, to avoid multicollinearity in the multivariate analyses, we conducted two series of analyses, one using BADL scores and the other using IADL scores as one of the predictors (Table 3). In the IADL model, a diagnosis of dementia was the most significant predictor for OT referral (OR = 61.49), followed by SPMSQ scores between 2 and 9 (OR = 14.60), joint ROM limitations (OR = 13.76), fear of falling (OR = 11.45), psychiatric disorders (OR = 11.04), and ≥ 2 behavioral or emotional problems, or both (OR = 9.87). An increase of 1 point in the Lawton IADL scale was associated with a 0.41 increase in the OR. The BADL model showed similar results: dementia was the most important predictor for OT referral (OR = 48.26), followed by ROM limitations (OR = 13.97), psychiatric disorders (OR = 13.95), fear of falling (OR = 13.89), SPMSQ scores between 2 and 9 (OR = 8.87 ability to express oneself (OR = 6.85), and ≥ 3 behavioral or emotional problems (OR = 5.79). An increase of 1 point in the Barthel Index was associated with a 0.77 increase in the OR (Table 3). The estimated areas under the curve (AUC) of the ROC curve for IADL and BADL models were significantly higher than expected: 0.977 and 0.972, respectively (Table 3).

To predict a client’s odds for OT referrals, we computed the estimated risk score $\hat{\eta }$ in two models defined by the following formulas [37]:

$$\begin{array}{l} {\hat{\eta }}_{\;\text{IADL}}=\text{logit}\;({\hat{\text{P}}}_{\text{i}})=\text{log}\left(\frac{{\hat{\text{P}}}_{\text{i}}}{1-{\hat{\text{P}}}_{\text{i}}}\right)=4.71-0.89\times (\text{IADL score})+2.29\times (\text{BPSD}\geq 2)+2.44\times (\text{Fear of falls})+\\ 2.68\times (\text{SPMSQ}.2-9)+4.12\times (\text{Dementia})+2.62\times (\text{ROM limitation}\;)+2.40\times (\text{Psychiatric diagnosis}) \end{array}$$

$$\begin{array}{l} {\hat{\eta }}_{\;\text{BADL}}=\text{logit}({\hat{\text{P}}}_{\text{i}})=\text{log}\left(\frac{{\hat{\text{P}}}_{\text{i}}}{1-{\hat{\text{P}}}_{\text{i}}}\right)=26.38-0.26\times (\text{BI score})+1.76\times (\text{BPSD}\geq 3)+2.63\times (\text{Fear of falls})+\\ 2.18\times (\text{SPMSQ}.2-9)+3.88\times (\text{Dementia})+2.64\times (\text{ROM limitation})+2.64\times (\text{Psychiatric diagnosis})+\\ 1.92\times (\text{Difficulty expressing oneself}\;) \end{array}$$

We next computed the estimated probability of an OT referral ($\hat{\text{P}}$), which is defined as:

$$\hat{\text{P}}=\frac{1}{1+{\text{exp}}^{-\hat{\eta }}}$$

Based on these results, two formulas, one for the IADL model, and one for the BADL model, can be used to calculate the probability of needing community-based OT service. Two client examples based on the IADL model are given in Table 4. The Hosmer-Lemeshow calibration tables for the BADL and IADL models are shown in Table 5. Both models perform very well. The number of participants being referred corresponds exactly with the predicted number of participants being referred beyond the 30% to 40% (the fourth decile) range in both models.

**Table 4:** Two Hypothetical Examples of the Probability of Needing Referral for Community-Based OT Based on the IADL Model of Referral Protocol.

Variable	Client 1	Client 2
**Age (years)**	91	61
**Gender**	M	F
**Stroke**	Yes	No
**Dementia**	No	No
**Psych Disorder**	Yes	No
**Lawton scale**	6	7
**SPMSQ**	9	9
**Behavioral / Emotional problems**	No	No
**Fear of falling**	Yes	Yes
**Limited ROM**	No	No
**Estimated risk score of Needing Referral for OT**^a^	1.0500	−1.5200
**Estimated Probability of Needing Referral for OT**^a^	0.7408	0.1795

OT: occupational therapy; IADL: instrumental activities of daily living; SPMSQ: Short Portable Mental Status Questionnaire, range: 0–10; ROM: range of motion.

^a^ The calculations were based on the variable values and the equations listed in the text.

**Table 5:** Hosmer-Lemeshow Calibration Table for the Basic Activities of Daily Living Model and the Instrumental Activities of Daily Living Model.

Predicted Probability	Observed Number of Participants Being Referred	Expected Number of Participants Being Referred	Predicted Probability	Observed Number of Participants Being Referred	Expected Number of Participants Being Referred
Basic Activities of Daily Living			Instrumental Activities of Daily Living		
0–0.1	3	2.88	0–0.1	0	0.89
0.1–0.2	5	3.54	0.1–0.2	6	4.43
0.2–0.3	12	14.12	0.2–0.3	13	13.87
0.3–0.4	19	18.77	0.3–0.4	20	20.30
0.4–0.5	22	21.72	0.4–0.5	22	21.59
0.5–0.6	22	21.97	0.5–0.6	22	21.93
0.6–0.7	22	22.00	0.6–0.7	23	22.98
0.7–0.8	22	22.00	0.7–0.8	23	23.00
0.8–0.9	23	23.00	0.8–0.9	21	21.00
0.9–1.0	21	21.00	0.9–1.0	21	21.00

## Discussion

We developed two referral protocol for the BADL and IADL models using the MDAI to predict which LTC clients in Taiwan should be referred for community-based OT services based upon the clinical judgments of occupational therapists. We found that dementia, psychiatric disorders, mild and moderate cognitive dysfunction, limited joint ROM, fear of falling, multiple behavioral or emotional problems, or both, and an inability to adequately express oneself (only in the BADL model) were the main factors associated with a need for OT referral. We constructed from our analysis two formulas to use for establishing a community-based OT referral threshold in Taiwan. Putting a client’s clinical data from the MDAI into the equations will provide the probability of being requiring community-based OT services in Taiwan. A threshold for OT referral can be set based on the available resources. For example, when resources are limited, the threshold can be set at 0.9 (the clients with probability higher than 0.9 will be referred), when there are more resources, at 0.7.

The high rate (77.4%) of need for OT referral in the study was likely due to the characteristics of the study participants. The participants, clients with mental or physical disabilities and BADL or IADL limitations, were already more likely to need OT services. We expect a lower referral rate if the sample is chosen from community-dwelling older adults. Potential clients of OT services were evaluated by experienced occupational therapists to determine if they will benefit from OT. However, the amount, extent, and intensity of OT services were not defined because we only asked the therapists to decide whether the clients needed OT. In fact, some clients may need only a consultation.

We found a high consistency (*κ* = 0.70) between the referrals made by occupational therapists who reviewed only the MDAI data completed by trained interviewers and the referrals made by occupational therapists who personally visited clients, which indicated that our models provided properly designed, objective measures for evaluating the need for OT services. Moreover, the very good predictive accuracy based on our regression model indicated that decisions made based on MDAI data were consistent with those made by OT experts. This finding provides an important clinical implication: when using an empirical referral protocol, care managers with proper training are able to identify clients who need OT services.

However, there were still some disagreements between two experts. Most disagreements arose when the occupational therapists were uncertain whether the therapy resources would induce a significant functional change. For example, the occupational therapists were uncertain about the effectiveness of preventive intervention and caregiver education for clients with a low potential for changes. Future criteria can be modified in accordance with regulations and available resources.

Both the BADL and the IADL models showed excellent fits and thus had good predictive accuracy. However, the BADL model is recommended as the first choice for healthcare professionals who routinely have to refer people for OT services, because BADL data are more commonly collected. IADL is normally used as a complementary index for measuring less severe levels of disability, including tasks that require a higher level of personal autonomy [5, 14, 19, 20]. Furthermore, IADL performance might be more closely related to social and cultural circumstances in different ethnic groups. Specifically, some people may not perform certain IADL tasks because they are not expected to do them or because they do not want to do them, not because they cannot do them. For example, elderly men are not expected to cook in some cultures. However, people with mental dysfunctions like mild dementia, chronic psychiatric disorders, and intellectual disabilities usually can independently perform their BADLs; thus, we suggest using the IADL model for predicting whether people without BADL limitations need OT services.

Our findings highlight the importance of referring clients with dementia for OT treatment. Alzheimer’s disease and other types of dementia are prevalent among older adults in LTC settings. For example, 26.7% of community-based LTC users in Taiwan [38] and 30.1% of community-dwelling older adults in the U.S. were diagnosed with dementia [5]. Five to ten home visits that provide OT for community-based people with dementia significantly improve their function, mood, QoL, health status, and sense of control over their lives, and they reduce the burden of the caregivers [39–42]. Thus, it was appropriate to refer some clients with dementia for a complete assessment.

Having more than 2 or 3 behavioral and emotional problems is related to OT referrals in both models. Adults with intellectual disabilities, autism spectrum disorders, and developmental disabilities frequently evince challenging behaviors [43, 44]. More than 70% of people with dementia have behavioral or psychological symptoms [45, 46] that are associated with poor life skills, jeopardize their health and safety, significantly exacerbate their caregiver’s burden, and decrease the QoL of both the client and the caregiver. These are the major reasons that many clients with dementia live in restrictive residential facilities rather than at home with family and caregivers [47, 48]. OT intervention based on sensory stimulation has efficaciously improved behavioral problems [49]. Thus, we believe that community-based OT will help clients and caregivers manage the clients’ behavioral and emotional problems.

It is interesting to note that the SPMSQ score was not linearly associated with the OT referral; that is, clients with SPMSQ scores between 2 and 9 were more likely to be recommended for OT services. Studies [27, 50] reported inconsistent conclusions about whether cognitive impairment is a significant negative predictor for rehabilitation potential and improvement in older patients [27, 50]. This might be because these studies used dichotomy scoring (i.e., asked whether the client had any impairment in cognitive skills for daily decision-making), which is not sensitive enough to discriminate between different levels of cognitive impairments. This finding is consistent with our clinical experience in which clients with a mild or moderate level of cognitive impairment usually required more OT intervention than did clients with no cognitive impairments and severe cognitive impairments. Clients with severe cognitive impairment (SPMSQ = 0 or 1) are usually severely limited in BADLs as well as IADLs, cannot follow verbal instructions, have rather low rehabilitation potentials, and, therefore, are less likely to be referred for OT services. Clients with intact cognitive function (i.e., SPMSQ = 10) are less likely to be ADL-dependent and thus less likely to need OT services.

Our study shows that a fear of falling was more associated with OT referrals than was falling within 6 months. Elderly clients with no history of falling often reported that they were afraid of falling [51]. This fear is not merely the result of the psychological trauma of falling [52]; it might also be a composite indicator of functional limitations related to physical, psychological, and environmental factors, and might predict BADL performance and mobility. People who are afraid of falling often voluntarily restrict their BADLs and any social participation that might impose a higher risk of functional decline and, subsequently, might require rehabilitation services [53]. OT intervention focuses not only on functional balance and mobility training, but also on the client’s confidence to participate in daily tasks without being afraid of falling. One systematic review of occupational therapy for community-dwelling older people (≧60) concluded that training them and conducting a home-hazard assessment decreases the incidence of falls for those with a high risk of falling [54]. Another study [55] emphasized that a home hazard assessment alone seemed inadequate to reduce falls or improve function, and a person-environment perspective is required for effective intervention to increase functionality in fall-related outcomes.

This prospective study analyzed the best predictors of community-based OT referrals and introduced empirical guidelines for community-based LTC rehabilitation service referrals. Only a few studies have investigated the correlates of “utilization” for community-based rehabilitation services rather than the correlates of the “need” for those services [27]. For example, using data from the 2005 National Health Interview Survey in Taiwan, a multivariate analysis in a population-based study [38] showed that age, being unmarried, and having a stroke, dementia, and an ADL disability are predictors for using community-based and institution-based LTC services. Our study empirically analyzed the correlates for OT referral by experienced occupational therapists rather than by other professionals or policy makers. It focused on whether the occupational therapists thought that the disabled client would benefit from the OT services rather than whether the client would actually use them. This distinction is important because many studies report that using LTC services might be affected by other factors, such as sociodemographics, manpower or financial resources, government subsidy regulations in different countries, and methods to access information [2] rather than the needs of the clients. Using correlates of utilization as indications for OT referral might not be appropriate.

The MDAI was designed for care managers to complete within a single visit. Several referral criteria considered important in OT focus groups were excluded in the final version by the MDAI development committee because the training and time required was not practical for care managers. These items included questions on daily routine, visual perceptual function, hand function, and length or history of disability. Thus, the MDAI cannot replace a comprehensive OT assessment. The purpose of identifying the predictive variables is to provide a referral protocol for care managers and to ensure an efficient and timely OT referral. These predictive variables, however, are not necessarily adequate substitutes for an in-person OT evaluation to determine whether an OT referral should be made.

We originally planned to conduct separate analyses for participants with mental or physical disorders since they were thought to have different etiologies and thus may have different indications for community-based OT services and different referral criteria. However, our analyses showed that both the BADL and IADL models had excellent model-fits (AUC = 0.972 and 0.977, respectively) for the combined sample. Therefore, it was not necessary to separate the participants for analysis. The results of these two predictive models include important predictors for people with mental and physical disorders. In fact, one of the difficulties of validating screening systems is the lack of a gold standard with which to compare [56]. The excellent model-fitting in our analyses is attributable to the clinical OT referral decisions made based on the MDAI, which contains all the predictors in our models. Thus, the outcome was based on clinical criteria, not on a natural outcome like death.

This study has some limitations. First, only 30% of the study pool was successfully interviewed. The remainder refused to participate, had died, were lost to follow-up, or had been admitted into LTC facilities. We were unable to make comparisons between the participants and the non-participants because of restrictions imposed by the Personal Information Protection Act, which prevented us from obtaining the data of non-participants. Second, this study relied on the self-reported health conditions of the participants, which might not accurately reflect their health status. Last, we classified the clients into only two groups: *Need for OT* and *No-need for OT*. In fact, some clients might have needed only a consultation. In the future, we will explore the possibility of using different levels of OT needs as the outcome variable in a triage model. Moreover, future studies are needed to confirm the efficacy of the referral protocol by examining its accuracy when compared with the initial OT assessment of their need for service. Cross-cultural comparison of the utility of the MDAI might also be useful.

## Conclusions

We developed two referral algorithms (the BADL and IADL models) based on the MDAI to predict which LTC clients in Taiwan should be referred for a comprehensive OT assessment. Both BADL and IADL models had very good predictive accuracy for the OT referral. The IADL model predicted which clients without BADL limitations needed OT services. These referral protocols should facilitate proper and timely decision-making for care managers to guide appropriate care planning and the allocation of limited resources in the LTC system. The methods and findings provided might also be useful for future studies by researchers interested in developing referral protocols for other LTC services.
